# Case report: Peritonitis caused by *Pantoea agglomerans* from pet dog exposure in peritoneal dialysis

**DOI:** 10.1097/MD.0000000000040247

**Published:** 2024-11-08

**Authors:** Ji Yeon Kim, Nam-Jun Cho, Samel Park, Hyo-Wook Gil, Eun Young Lee

**Affiliations:** a Department of Medicine, College of Medicine, Soonchunhyang University, Cheonan, Korea; b Department of Internal Medicine, Soonchunhyang University, Cheonan Hospital, Cheonan, Korea; c BK21 Four Project, College of Medicine, Soonchunhyang University, Cheonan, Korea; d Institute of Tissue Regeneration, College of Medicine, Soonchunhyang University, Cheonan Hospital, Cheonan, Korea.

**Keywords:** *Pantoea agglomerans*, peritoneal dialysis, peritonitis, pet dog

## Abstract

**Rationale:**

Peritonitis caused by *Pantoea agglomerans* is a rare occurrence in patients undergoing peritoneal dialysis. Cases potentially linked to pet dogs are even rarer, and there is limited literature available.

**Patient concerns:**

A patient undergoing peritoneal dialysis presented with symptoms of peritonitis, including abdominal pain and cloudy dialysis fluid.

**Diagnoses:**

Microbiological analysis identified *P agglomerans* as the causative organism.

**Interventions:**

The patient was treated with targeted antibiotic therapy and showed a positive response.

**Outcomes:**

During a subsequent medical interview, it was revealed that the patient had close contact with their pet dog, raising the possibility that the infection may have been associated with this exposure.

**Lessons:**

This case highlights the importance of considering zoonotic transmission as a potential source of infection in peritoneal dialysis patients, particularly when there is close contact with pets. Healthcare providers should educate patients about the potential risks posed by pets and implement preventive strategies to mitigate such risks.

## 1. Introduction

Although peritonitis is a common complication in patients undergoing peritoneal dialysis, cases caused by *Pantoea agglomerans* are rare, with only 12 reported worldwide since its identification in 2005.^[1]^ *Pantoea agglomerans*, belonging to the Enterobacteriaceae family, is frequently found in plants like palm trees, cacti, and rose bushes, as well as in animal excreta.^[2,3]^ Most reported cases of *P agglomerans*-related peritonitis in peritoneal dialysis patients have been attributed to close contact with plants, such as during gardening.^[1]^ While rare, 1 case has been reported involving close contact with pet dogs.^[4]^ This report describes a case of peritonitis caused by *P agglomerans* acquired through contact with a pet dog, emphasizing the potential for animal-related infections in such cases. We also review 4 additional reported cases of dog-related peritonitis, contributing to the understanding of this rare but important association.

## 2. Case description

**Figure 1.**
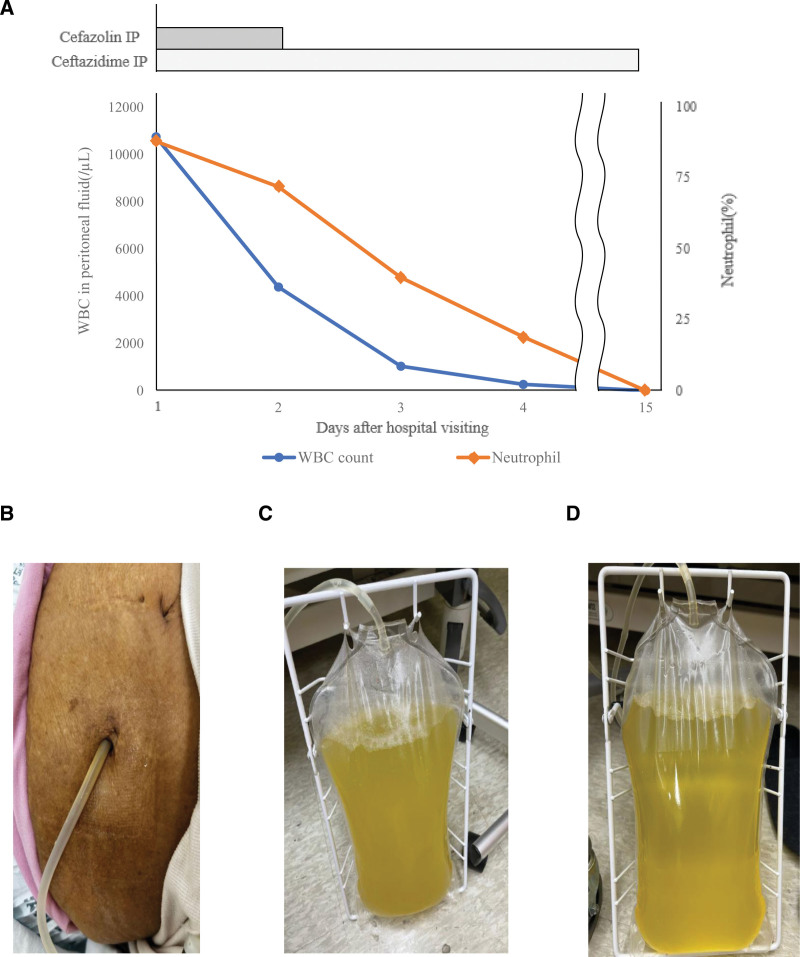
Patient’s clinical course. (A) Patient’s clinical progress. Both the leukocyte count and neutrophil ratio in the peritoneal fluid have decreased. On day 2 after hospital visit, based on interim report findings, cefazolin was discontinued, and only ceftazidime was continued. On day 15 after the hospital visit, the leukocyte count decreased to 4, leading to discontinuation of antibiotic therapy. (B) Clean exit site of the peritoneal dialysis catheter, no discharge detected. (C) Peritoneal fluid drawn on the day of admission. (D) Peritoneal fluid obtained on day 3 of hospitalization, showing improved turbidity.

A 76-year-old female patient, with a history of chronic kidney disease due to hypertension and undergoing peritoneal dialysis since 2008, presented to the emergency room on May 15, 2023, with severe abdominal pain. Initially, she experienced lower abdominal pain, but it progressed to a cramp-like, colicky pain involving the entire abdomen, rated 10 on the numerical rating scale. The patient’s vital signs at admission were as follows: blood pressure 100/60 mm Hg, heart rate 96 beats per minute, respiratory rate 30 breaths per minute, and body temperature 38.3°C. Physical examination revealed diffuse abdominal tenderness, rebound tenderness, and abdominal rigidity, though the exit site of the peritoneal dialysis catheter was clean, with no signs of discharge (Fig. 1B).

Given the patient’s history and symptoms, an infectious cause was prioritized. A sample of the peritoneal dialysis fluid was collected for analysis, revealing a white blood cell count of 10,745/µL, with 88% polymorphonuclear neutrophils, 2% lymphocytes, 9% macrophages, and 1% mesothelial cells. The peritoneal fluid appeared pale yellow and turbid (Fig. 1C). Based on these findings, a diagnosis of bacterial peritonitis was made. The patient was started on empirical intraperitoneal antibiotics, specifically cefazolin and ceftazidime.

On the second day of hospitalization, the patient’s abdominal pain began to improve, but the peritoneal fluid remained turbid. A preliminary report from the peritoneal fluid culture showed gram-negative bacilli, prompting the discontinuation of cefazolin and continuation of ceftazidime. By the third day, *P agglomerans* was isolated from the culture. A susceptibility test showed that the organism was sensitive to ceftazidime, and treatment with this antibiotic was continued. The WBC count in the peritoneal fluid decreased, and the turbidity improved gradually (Fig. 1D).

Further investigation into the source of infection revealed no history of plant cultivation or close contact with plants, which are typical sources of *P agglomerans*. However, during a detailed interview, it was discovered that the patient had a pet dog that was frequently allowed access to the room where peritoneal dialysis took place. This raised the possibility of zoonotic transmission through contact with the dialysis catheter.

The patient responded well to antibiotic therapy. After completing training on self-injection of antibiotics, she was discharged on May 26, 2023. At the time of discharge, her symptoms had significantly improved, and follow-up testing showed a marked decrease in the WBC count in the peritoneal fluid. Eleven days later, at a follow-up visit, the WBC count had normalized to 4/µL, and the antibiotic treatment was discontinued. The patient’s clinical course is summarized in the table and figure below (Table 1, Fig. 1A). Preventive measures were implemented, including restricting the dog’s access to the dialysis area and reinforcing hygiene protocols. No recurrent infections have been reported since the initial episode.

**Table 1:** Outline of clinical progression in a case.

Date	Event	Notes
May 15, 2023	Patient presented to the ER with severe abdominal pain	Initial symptom: cramp-like pain, NRS 10
May 15, 2023	Diagnostic tests revealed bacterial peritonitis	Peritoneal fluid WBC count: 10,745/µL, antibiotics initiated
May 16, 2023	Symptoms improved, but peritoneal fluid remained turbid	Empiric therapy with cefazolin discontinued; ceftazidime continued
May 17, 2023	*Pantoea agglomerans* cultured from peritoneal fluid	Continued treatment with ceftazidime
May 26, 2023	Patient discharged with self-injection training	WBC count improved, no recurrent infections
June 6, 2023	Follow-up visit with complete resolution of infection	Final WBC count: 4/µL, antibiotic therapy discontinued

NRS = numerical rating scale, WBC = white blood cell.

This case highlights the potential risk of peritonitis related to pet contact in peritoneal dialysis patients, particularly in those with close proximity to animals. The need for patient education on maintaining strict hygiene and limiting pet access in such settings is crucial to prevent similar infections. This case serves as a reminder to consider zoonotic sources in cases of unexplained peritonitis.

## 3. Discussion

We report a case of peritonitis caused by *P agglomerans* in a peritoneal dialysis patient, likely due to close contact with a pet dog. This is the first such case reported in South Korea and only the second worldwide.^[1]^ Several rare cases of peritonitis caused by zoonotic organisms related to pet dogs have been reported in peritoneal dialysis patients.^[4–8]^ A total of 4 cases involving *P agglomerans*,^[4]^ *Pasteurella multocida*,^[5,6,8]^ and *Staphylococcus pseudintermedius*^[7]^ have been documented in dog owners undergoing peritoneal dialysis. Including our case, this brings the total to 5 reported cases of peritonitis associated with pet dogs (Table 2).

**Table 2:** Summary of published cases of dog-related peritonitis in peritoneal dialysis.

Case	Age/sex	ESKD cause	Dialysis vintage	Peritoneal culture organism	Dog exposure	Antibiotic used/route	Comments
2007^[5,8]^	48/F	Hypertension, Type 2 diabetes	NA	*Pasteurella multocida*	O	Genta and Cefa/IP	Presumably dog-related, in house
2008^[4]^	52/F	Obstructive uropathy	NA	*Pantoea agglomerans*	O	Cipro/Oral	Close contact with a dog
2013^[6,8]^	49/M	Type 1 diabetes	1 yr	*Pasteurella multocida*	O	Cefta/IP Amoxi/Oral	Pet exposure, direct inoculation unknown
2020^[7]^	39/F	Hypertension	8 wk	*Staphylococcus pesudintermedius*	O	Vanco and Pipera/IV	Dog sleeps with the patient
Our case	76/F	Hypertension	15 yr	*Pantoea agglomerans*	O	Cefta/IP	Dog freely enters PD room

Amoxi = amoxicillin-clavulanate, Cefa = cefazolin, Cefta = ceftazidime, Cipro = ciprofloxacin, ESKD = end-stage kidney disease, Genta = gentamicin, IP = intraperitoneal, IV = intravenous, NA = not available, PD = peritoneal dialysis, Pipera = piperacillin-tazobactam, Vanco = vancomycin.

A detailed review of the reported cases reveals that *Pasteurella multocida* was identified in cultures from 2 patients, while *P agglomerans* and *Staphylococcus pseudintermedius* were each isolated in 1 patient. The most common etiological agents in peritoneal dialysis-related peritonitis are *Staphylococcus aureus*, *Staphylococcus epidermidis*, and *Pseudomonas* species.^[1]^ A comparative analysis of zoonotic organisms involved in peritonitis related to animal contact, such as *Pasteurella spp.*, *Bordetella bronchiseptica*, *Capnocytophaga spp.*, *Brucella spp.*, and *Neisseria spp.*,^[9]^ suggests variability in the pathogens responsible. This highlights the potential for a wide range of zoonotic bacteria to cause human infections via exposure to pet skin or excreta.

Despite this diversity of potential pathogens, antibiotic regimens commonly used to treat general peritonitis have yielded favorable outcomes, as seen in our case and others. This suggests that standard antibiotic therapies remain effective even in cases involving zoonotic organisms. However, this case report underscores the need for heightened awareness regarding the risk of zoonotic infections in peritoneal dialysis patients, particularly with the rise in pet dog ownership and the absence of established patient education guidelines on pet contact and disease management.

While this report contributes to the growing body of literature on peritonitis in peritoneal dialysis patients associated with pets, there are limitations to consider. First, not all relevant cases could be included as the report is based on previously published case reviews. Second, this is a retrospective analysis of a single case from our institution, limiting broader generalizations. Thus, further studies are needed to assess the full scope of zoonotic risks and preventive strategies in this population.

In conclusion, this case highlights the importance of educating peritoneal dialysis patients about the potential risks posed by close contact with pets, particularly dogs. A thorough evaluation of patients’ medical history, including pet ownership and exposure, is crucial for effective prevention and management of peritonitis. By raising awareness and promoting preventive measures, we can help reduce the risk of infection in this vulnerable patient population.

## Author contributions

**Conceptualization:** Eun Young Lee.

**Writing – original draft:** Ji-Yeon Kim.

**Writing – review & editing:** Ji-Yeon Kim, Nam-Jun Cho, Samel Park, Hyo-Wook Gil, Eun Young Lee.

## References

[1] Mateus C Martins AR Toscano C Matias P Branco P. Pantoea in peritoneal dialysis: a rare cause of peritonitis. Cureus. 2022;14:e26878. 35978740 10.7759/cureus.26878 PMC9375850

[2] Lim PS Chen SL Tsai CY Pai MA. Pantoea peritonitis in a patient receiving chronic ambulatory peritoneal dialysis. Nephrology (Carlton). 2006;11:97–9. 16669968 10.1111/j.1440-1797.2006.00552.x

[3] Kratz A Greenberg D Barki Y Cohen E Lifshitz M. *Pantoea agglomerans* as a cause of septic arthritis after palm tree thorn injury; case report and literature review. Arch Dis Child. 2003;88:542–4. 12765929 10.1136/adc.88.6.542 PMC1763127

[4] Habhab W Blake PG. Pantoea peritonitis: not just a “thorny” problem. Perit Dial Int. 2008;28:430. 18556396

[5] Antony SJ Oglesby KA. Peritonitis associated with *Pasteurella multocida* in peritoneal dialysis patients – case report and review of the literature. Clin Nephrol. 2007;68:52–6. 17703837 10.5414/cnp68052

[6] Al-Fifi YS Sathianathan C Murray BL Alfa MJ. Pets are “risky business” for patients undergoing continuous ambulatory peritoneal dialysis. Can J Infect Dis Med Microbiol. 2013;24:e96–8. 24421840 10.1155/2013/829534 PMC3852466

[7] Dahbour L Gibbs J Coletta C. Peritoneal dialysis zoonotic bacterial peritonitis with *staphylococcus pseudintermedius*. Case Rep Nephrol Dial. 2020;10:65–70. 32775342 10.1159/000508126 PMC7383182

[8] Giacona JM Weiner M Hanna J Jodlowski T Bedimo R. Pasteurella multocida bacteremia secondary to peritoneal dialysis associated peritonitis: a case report and literature review. Cureus. 2022;14:e24188. 35592208 10.7759/cureus.24188 PMC9109734

[9] Broughton A Verger C Goffin E. Pets-related peritonitis in peritoneal dialysis: companion animals or trojan horses? Semin Dial. 2010;23:306–16. 20636924 10.1111/j.1525-139X.2010.00726.x

